# Toward Retrieval-Grounded Evaluation for Conversational Large Language Model–Based Risk Assessment

**DOI:** 10.2196/90759

**Published:** 2026-03-12

**Authors:** Yihan Hu

**Affiliations:** 1 MRC Epidemiology Unit, University of Cambridge, The Old Schools, Trinity Ln, Cambridge, England, CB2 1TN, United Kingdom, 44 07526543793

**Keywords:** personalized risk assessment, large language model, artificial intelligence, conversational AI, COVID-19

We read with great interest the paper by Roshani et al [1] on a generative large language model (LLM)–powered conversational app for pediatric COVID-19 severity risk assessment. The study makes a timely and valuable contribution by demonstrating how white-box LLMs (LLaMA2 [Meta], T0 [BigScience], and Flan-T5 [Google]) can be fine-tuned in low-data settings and deployed within an end-to-end mobile workflow. We also commend the authors for emphasizing local deployment and interpretability through attention-based feature importance.

We would like to highlight one methodological consideration that may affect how readers interpret the reported accuracy and the system’s readiness for clinical or public-facing use. In the study, model outputs are operationalized as a binary risk score derived from the token probabilities of “yes” versus “no,” and performance is primarily summarized using the area under the receiver operating characteristic curve (AUC) [1]. While this is an appropriate discriminative metric for outcome prediction within the dataset, the system is presented as a conversational interface intended to support real-time risk assessment. In such settings, a key safety concern extends beyond misclassification to the generation of fluent but unverifiable or incorrect clinical statements. Recent empirical evaluations of retrieval-augmented LLMs in guideline-grounded clinical tasks underscore the importance of grounding and safety-focused assessment in medical settings [2].

Recent advances in retrieval-augmented generation (RAG) challenge the implicit assumption that response fluency and stable token-level scoring sufficiently capture clinical reliability. Under an LLM-only paradigm, outputs may appear consistent and plausible, yet factual correctness is difficult to audit in the absence of explicit source grounding. By contrast, RAG enables models to condition responses on external authoritative resources, such as clinical guidelines or curated medical databases, thereby supporting source-linked verification and more stringent evaluation [3]. Importantly, this represents not merely an architectural refinement but a shift in what can be meaningfully evaluated.

We therefore suggest that future work in this area would benefit from a retrieval-grounded sensitivity analysis alongside conventional AUC reporting. For example, the same conversational pipeline could be evaluated under two conditions: (1) the current LLM-only setting and (2) an evidence-grounded setting in which responses are generated with explicit citations to a prespecified clinical corpus, such as pediatric COVID-19 guidance from the Centers for Disease Control and Prevention or equivalent institutional protocols. Evaluation could then incorporate evidence-grounded correctness, citation faithfulness, and robustness to retrieval constraints. In addition, subgroup-level audits—using metrics such as recall or *F*_1_-score stratified by demographic or social determinant variables—could help identify whether aggregate performance masks safety-relevant disparities across populations.

We acknowledge that retrieval-grounded approaches introduce practical challenges, including retrieval latency and the need for ongoing corpus curation. Nonetheless, in high-stakes pediatric and public health applications, these trade-offs may be justified by gains in verifiability and trustworthiness. We commend the authors for advancing conversational LLM–based risk assessment and hope these considerations help further strengthen evaluation rigor and clinical interpretability in future deployments.

## Abbreviations

AUC: area under the receiver operating characteristic curve
LLM: large language model
RAG: retrieval-augmented generation
